# Omega-3 fatty acids and individual variability in plasma triglyceride response: A mini-review

**DOI:** 10.1016/j.redox.2023.102730

**Published:** 2023-05-03

**Authors:** Amanda Rundblad, Viviana Sandoval, Kirsten B. Holven, José M. Ordovás, Stine M. Ulven

**Affiliations:** a Department of Nutrition, Institute of Basic Medical Sciences, University of Oslo, P.O Box 1046 Blindern, 0317, Oslo, Norway; b Escuela de Nutrición y Dietética, Facultad de Ciencias para el Cuidado de la Salud, Universidad San Sebastián, Gral. Lagos 1025, 5110693, Valdivia, Chile; c Norwegian National Advisory Unit on Familial Hypercholesterolemia, Oslo University Hospital, Norway; d Nutrition and Genomics Laboratory, USDA ARS, JM-USDA Human Research Center on Aging at Tufts University, Boston, MA, USA; e Nutritional Genomics and Epigenomics Group, Precision Nutrition and Obesity Program, IMDEA Food, CEI UAM + CSIC, Madrid, Spain; f Centro de Investigación Biomédica en Red Fisiopatología de la Obesidad y la Nutrición (CIBEROBN), Institute of Health Carlos III, Madrid, Spain

**Keywords:** Triglycerides, Omega-3 fatty acids, Genotype, Epigenetics, Gene expression, Gut microbiota

## Abstract

Cardiovascular disease (CVD) is a leading cause of death worldwide. Supplementation with the marine omega-3 fatty acids eicosapentaenoic acid (EPA) and docosahexaenoic acid (DHA) is associated with lower CVD risk. However, results from randomized controlled trials that examine the effect of omega-3 supplementation on CVD risk are inconsistent. This risk-reducing effect may be mediated by reducing inflammation, oxidative stress and serum triglyceride (TG) levels. However, not all individuals respond by reducing TG levels after omega-3 supplementation. This inter-individual variability in TG response to omega-3 supplementation is not fully understood. Hence, we aim to review the evidence for how interactions between omega-3 fatty acid supplementation and genetic variants, epigenetic and gene expression profiling, gut microbiota and habitual intake of omega-3 fatty acids can explain why the TG response differs between individuals. This may contribute to understanding the current controversies and play a role in defining future personalized guidelines to prevent CVD.

## Highlights

• Omega-3 fatty acids may lower CVD risk by reducing serum TG levels.
• However, not all individuals respond to omega-3 supplementation by lowering the TG levels.
• Different participant characteristics may contribute to this inter-individual variability in TG response.
• Genetic polymorphisms seems to be most important to explain this variation.

## Graphical abstract

Genetic variants, epigenetic modifications, miRNAs, gene expression profiles, gut microbiota and the habitual intake of omega-3 fatty acids may influence the TG response to omega-3 supplementation. Illustrations from Servier Medical Art and Colourbox.

**Figure:**
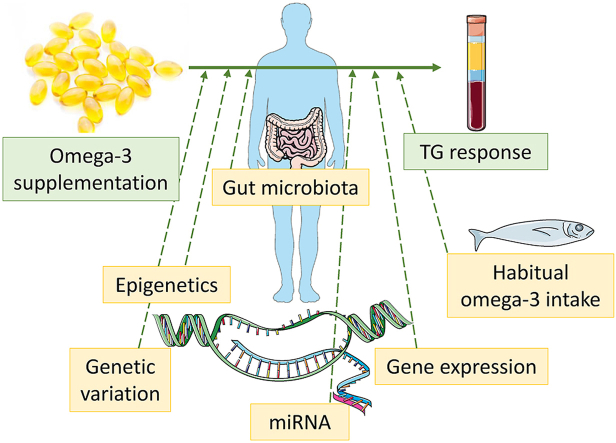

## 1 Introduction

Intake of fish containing the marine long chain omega-3 fatty acids EPA and DHA is an important component of a healthy diet to prevent lifestyle diseases [1]. To ensure availability of these fatty acids if dietary intakes are low, they can be endogenously produced from the essential fatty acid alpha-linolenic acid through a series of reactions where fatty acid desaturase (FADS) 1 and 2 are the rate-limiting enzymes [2]. During human evolution, a derived haplotype of the *FADS* gene cluster associated with more efficient production of long chain polyunsaturated fatty acids (LC-PUFA) appeared [3]. While selection of the derived haplotype was favored in populations that started cultivating the soil, selection of the haplotype with the least efficient desaturases was favored in populations with a high intake of LC-PUFA from marine sources [4,5].

Today, CVD is one of the leading causes of death worldwide [1]. Supplementation with omega-3 fatty acids may lower risk for coronary heart disease (CHD) events and mortality, as well as total CVD and CVD death [6,7]. However, results from randomized controlled trials (RCTs) that examine the effect of omega-3 fatty acids on CVD risk are inconsistent [8]. Some of these conflicting results may be due to study design differences, such as patient populations, omega-3 formulation, dose, placebo treatment, background diet, baseline statin use, and follow-up duration [8,9]. For example, the VITAL trial did not find a reduced incidence of major cardiovascular events after intake of 840 mg EPA + DHA/day with a 5.3-year median follow-up. However, a subset of participants with low habitual fish consumption had reduced CVD risk, suggesting that the effect of marine omega-3 fatty acids on CVD risk may depend on the habitual diet [10]. Moreover, the STRENGTH and REDUCE-IT trials were RCTs with the same omega-3 dose. The STRENGTH trial (4 g EPA + DHA/d) resulted in no significant difference in major adverse cardiovascular events between the groups, despite a 19% TG reduction [11]. Finally, the REDUCE-IT trial (4 g EPA/d) found that the risk of ischemic events, including cardiovascular death, was significantly lower in the omega-3 group compared to placebo, which suggests that there are possible distinct effects of DHA that counteract the benefits of EPA [12].

Omega-3 fatty acids are prone to oxidation because of their high degree of unsaturation. This has led to concern about intake of potentially oxidized omega-3 supplements. However, oxidized fish oil does not seem to increase plasma or urine levels of markers of oxidative stress, lipid peroxidation, inflammation or oxidized low-density lipoprotein (LDL) [13,14]. Nonetheless, oxidized fish oil may have a detrimental effect on the concentration of LDL subclasses [15]. Contrary to the concern about omega-3 fatty acids’ detrimental effect on oxidative stress, intake of omega-3 fatty acids, in particular EPA, may protect against oxidation of LDL [[16], [17], [18]].

**Fig. 1:**
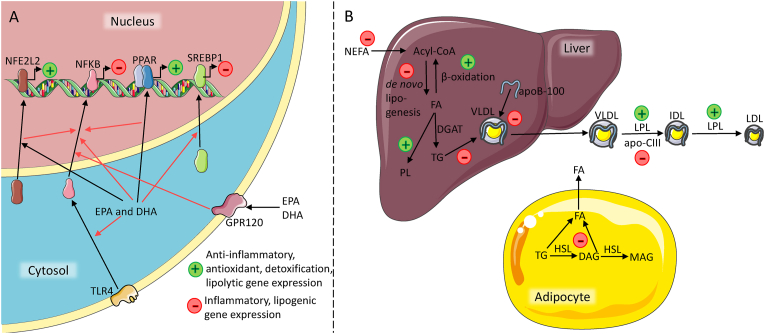
**A)** EPA and DHA alter gene expression of genes involved in antioxidant response, detoxification and inflammation by activating NFE2L2 and inhibiting NFKB via activation of PPARs, inhibition of TLR4 signaling and through inhibition via GPR 120. Moreover, EPA and DHA alter expression of genes involved in lipid metabolism by activating PPARs and inhibiting expression and nuclear translocation of SREBP1. **B)** EPA and DHA reduce plasma TG levels by reducing VLDL synthesis and *de novo* lipogenesis by inhibiting DGAT. Additionally, EPA and DHA increase fatty acid β-oxidation, reduce hepatic delivery of NEFA, and increase hepatic PL synthesis rather than TG. Illustrations from Servier Medical Art. Abbreviations: apoB-100, apolipoprotein-100, apo-CIII, apolipoprotein-CIII, DAG, diacylglycerol, DGAT, diacylglycerol O-acyltransferase, FA, fatty acid, GRP120, G-protein coupled receptor 120, HSL, hormone sensitive lipase, LPL, lipoprotein lipase, MAG, monoacylglycerol, NEFA, non-esterified fatty acids, NFKB, nuclear factor-kappa B, NFE2L2, NFE2 like bZIP transcription factor 2, PPAR, peroxisome proliferator-activated receptor, PL, phospholipids, SREBP, sterol regulatory element binding protein, TG, triglyceride, TLR4, toll-like receptor 4, VLDL, very low-density lipoprotein.

EPA and DHA may lower CVD risk through several different cellular effects, including altering gene expression of genes involved in oxidative stress, inflammation, and lipid metabolism (Fig. 1A). Firstly, omega-3 fatty acids may activate the transcription factor NFE2 like bZIP transcription factor 2 (NFE2L2) that induce the expression of anti-inflammatory genes and genes that encode antioxidant and detoxification enzymes, and thereby contribute to maintain redox homeostasis [19]. This mechanism may explain why omega-3 fatty acid supplementation increases serum total antioxidant capacity and glutathione peroxidase, and decrease malondialdehyde compared to placebo in human RCTs [20]. Secondly, omega-3 fatty acids may also reduce nuclear factor-kappa B (NFKB) induced expression of inflammation-related genes, partly via NFE2L2 activation, but also via activation of peroxisome proliferator-activated receptors (PPARs), inhibition of toll-like receptor 4 signaling by modulation of lipid rafts and through inhibition via G-protein coupled receptor 120 [19,[21], [22], [23]]. Finally, omega-3 fatty acids increase the expression of lipolytic genes by binding to and activating PPARs, and by inhibiting expression and nuclear translocation of sterol regulatory binding protein 1 (SREBP1), thus reducing the expression of lipogenic genes [24]. This effect on expression of genes involved in lipid metabolism may be important for the TG-reducing effect of omega-3 fatty acids.

**Fig. 2:**
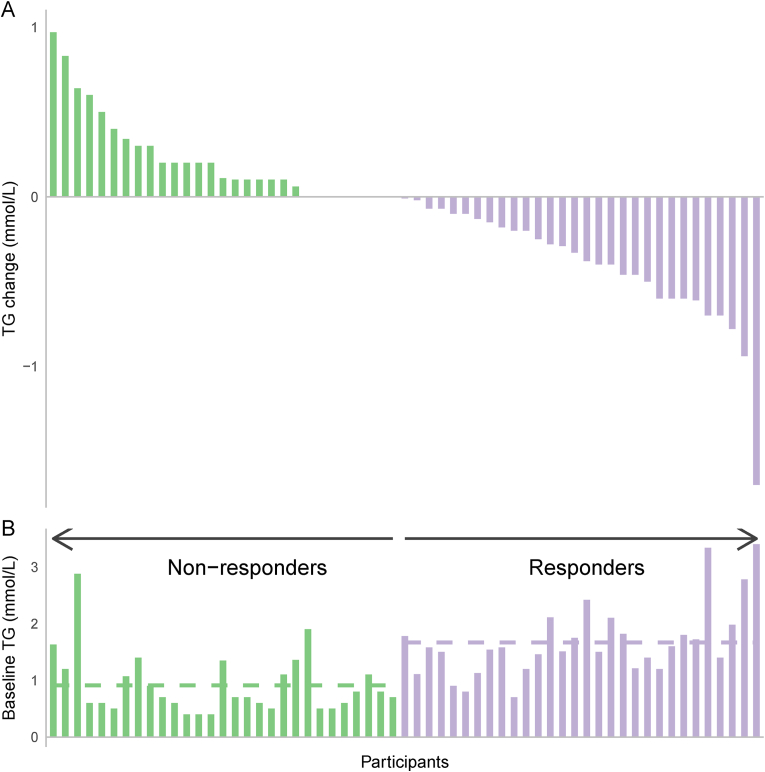
**A)** Individual variation in TG response to omega-3 supplementation. **B)** Baseline TG concentration is on average higher among those who respond to omega-3 supplementation by lowering TG concentrations. Dashed lines indicate mean baseline TG in non-responders (green) and responders (purple). Figure shows individual data from participants receiving omega-3 supplementation in two different randomized controlled trials [14,77]. (For interpretation of the references to colour in this figure legend, the reader is referred to the Web version of this article.)

The CVD risk-reducing effect of EPA and DHA is in part mediated by the reduction of serum TG levels, as elevated TG is strongly associated with an increased risk of CVD [25]. In a meta-analysis of large RCTs, subgroup analyses showed that subjects with elevated TG levels had reduced CVD risk after omega-3 supplementation [26]. EPA and DHA are potent dietary signaling molecules with well-described mechanisms for reducing plasma TG levels (Fig. 1B). These include reduced hepatic very low-density lipoprotein (VLDL) synthesis and *de novo* lipogenesis, caused by reduced fatty acid availability for TG synthesis due to blockade of diacylglycerol O-acyltransferase (DGAT), as well as increased fatty acid β-oxidation. This increased β-oxidation also leads to increased mitochondrial uncoupling and consequently reduced inflammation and production of reactive oxygen species [27]. However, to our knowledge, reduced inflammation and reactive oxygen species production do not seem to alter fasting TG levels. Finally, EPA and DHA reduce TG levels through reduced delivery of non-esterified fatty acids (NEFA) to the liver and increased hepatic synthesis of phospholipids rather than TG [28]. It is well known that some individuals respond to omega-3 supplementation by reducing TG levels, while others do not (Fig. 2 A) [[29], [30], [31], [32], [33]]. The inter-individual variability in TG response to omega-3 supplementation is not fully understood. However, participant characteristics such as genetic variants, epigenetic and gene expression profiles, gut microbiota and composition of the habitual diet may contribute to this variability [29].

With this in mind, we aim to review the evidence for how interactions between omega-3 fatty acid supplementation and genetic variants, epigenetic and gene expression profiling, gut microbiota and the habitual intake of omega-3 fatty acids can explain why the TG response differs between individuals (**Graphical abstract**). This information will contribute to understanding the current controversies and may play a role in defining future personalized guidelines for omega-3 fatty acid recommendations to prevent CVD.

## 2 Genotype variants and TG response to omega-3 fatty acid supplementation

**Fig. 3:**
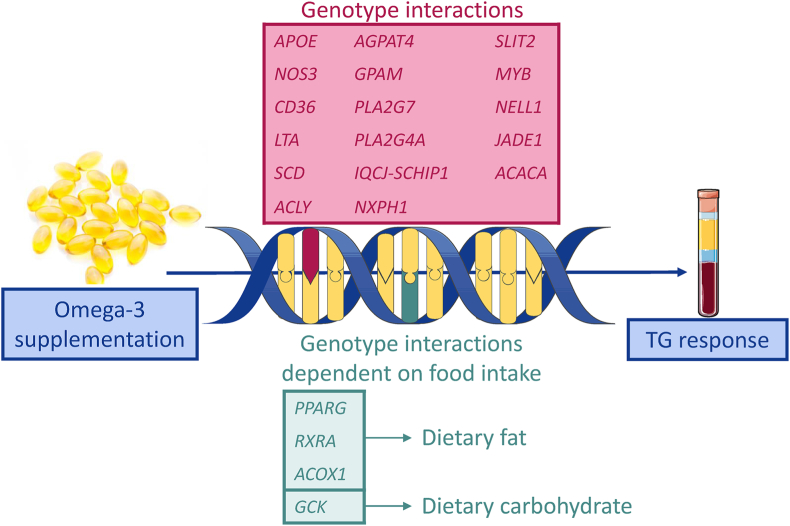
The TG response to omega-3 supplementation is associated with several genotypes, including genotype associations that are dependent on food intake. Abbreviations are explained in the list of abbreviations.

Single-nucleotide polymorphisms (SNPs) are single nucleotide variants (SNVs) in DNA present in at least 1% of the population. Such variations are useful to explain health, disease, drug response, and other inter-individual traits. Several studies have found genotypes that interact with the TG response to omega-3 supplementation (Table 1, Fig. 3). The TG response after omega-3 supplementation has been shown to interact with sex and apolipoprotein E (*APOE*) genotype, with ApoE4 males having the most significant TG reduction [32]. Moreover, SNPs in nitric oxide synthase 3 (*NOS3*), CD36 molecule (*CD36*) and lymphotoxin alpha (*LTA*, also known as TNFβ) are associated with the TG response to omega-3 supplementation [[34], [35], [36], [37]]. In a Canadian study, 208 participants were supplemented with 5 g fish oil dayily (∼2 g EPA + 1 g DHA) for 6 weeks. SNPs in genes involved in lipid metabolism (stearoyl-CoA desaturase (*SCD*)*,* ATP citrate lyase (*ACLY*), acetyl-CoA carboxylase alpha (*ACACA*), 1-acylglycerol-3-phosphate O-acyltransferase 4 (*AGPAT4*), glycerol-3-phosphate acyltransferase, mitochondrial (*GPAM*), phospholipase A2 group VII (*PLA2G7*), and phospholipase A2 group IVA (*PLA2G4A*)), neuron function (IQCJ-SCHIP1 readthrough (*IQCJ-SCHIP1*), neurexophilin 1 (*NXPH1*) and slit guidance ligand 2 (*SLIT2*)), transcription (MYB proto-oncogene, transcription factor (*MYB*)), and cell growth (neural EGFL like 1 (*NELL1*) and jade family PHD finger 1 (*JADE1*)), had a significant genotype-supplementation interaction effect on the TG response [[38], [39], [40], [41], [42], [43]]. Some of these SNPs had a genotype frequency that differed between TG-responders and non-responders [39,42,43].

**Table 1:** Summary of studies that have investigated associations between the TG response to omega-3 supplementation and genotype, epigenetics, gene expression, non-coding RNA, gut microbiota and habitual diet.

Author	Ref	Summary of findings
**Genotype association with TG response to omega-3**		
Caslake et al., 2008	[32]	Greater TG reduction following omega-3 supplementation in male *APOE4* carriers compared to E2 and E3 carriers and females.
Ferguson et al., 2010	[34]	Plasma TG concentrations of minor allele carriers (AC + AA) of the *NOS3* rs1799983 SNP were more responsive to changes in plasma n-3 PUFA, than major allele (CC) homozygotes after omega-3 supplementation.
Madden et al., 2008	[35]	Significant TG reduction following omega-3 supplementation only occurred in individuals with the GG variant of the 25444G > A, 30294G > C, 31118G > A and 33137A > G SNPs of the *CD36* gene
Miao et al., 2022	[36]	Omega-3 exposure in the top quartile was associated with lower TG concentrations compared to the bottom quartile among *CD36* rs1527483-GG carriers, but not in AA/AG carriers.
Marcovic et al., 2004	[37]	Across all BMI tertiles, TG reduction after omega-3 supplementation occurred only in individuals possessing the AA genotype in position +252 of *LTA*, encoding TNFβ.
Rudkowska et al., 2014	[42]	Thirteen loci, including SNPs in or near *IQCJ-SCHIP1*, *MYB*, *NELL1*, *NXPH1*, *JADE1*, and *SLIT2*, had frequency differences between TG responders and non-responders to omega-3 supplementation. A genetic risk score (GRS) composed of these risk alleles explained 21.53% of the variation in TG response to omega-3 supplementation after adjusting for age, sex, and BMI. The GRS explained only 2% of TG variation in a replication cohort.
Bouchard-Mercier et al., 2013	[39]	Two SNPs, *ACLY* rs8071753 and *ACACA* rs1714987, were associated with TG change after omega-3 supplementation, and genotype frequencies of rs8071753 differed between TG responders and non-responders to omega-3 supplementation.
Ouellette et al., 2013	[40]	The plasma TG response to omega-3 supplementation was dependent on genotype for rs2792751 and rs17129561 in *GPAM* and rs3798943 and rs9458172 in *AGPAT4*.
Tremblay et al., 2015	[41]	The plasma TG response to omega-3 supplementation was dependent on genotype for rs1805018 in *PLA2G7* and rs10752979, rs10737277, rs7540602, and rs3820185 in *PLA2G4A*.
Rudkowska et al., 2014	[38]	The plasma TG response to omega-3 supplementation was dependent on genotype for rs508384 in *SCD.*
Vallée Marcotte et al., 2016	[43]	The plasma TG response to omega-3 supplementation was dependent on genotype for ten SNPs of *IQCJ*, four SNPs of *NXPH1* and three SNPs of *MYB*. TG responders and non-responders to omega-3 supplementation had different genotype frequencies with nine SNPs of *IQCJ*, two SNPs of *NXPH1* and two SNPs of *MYB*.
Vallée Marcotte et al., 2019	[44]	A GRS with 31 SNPs explained 49.73% of the variation in TG response to omega-3 supplementation, adjusted for age, sex, and body mass index. The GRS explained 3.67% of the TG response variation in a replication cohort. TG non-responders to omega-3 supplementation had a higher GRS than responders.
Vallée Marcotte et al., 2020	[45]	The same GRS as in Ref. [44] predicted TG response after EPA supplementation, and non-significantly for DHA supplementation.
Lindi et al., 2003	[46]	The plasma TG response to omega-3 supplementation was dependent on the Pro12Ala polymorphism of the *PPARG* gene. Carriers of the Ala12 allele had a greater TG reduction after omega-3 supplementation than the Pro12Pro genotype when dietary fat intake was lower than 37 E% or when dietary saturated fat intake was lower than 10 E%.
Bouchard-Mercier et al., 2014	[47]	The TG response to omega-3 supplementation was dependent on the rs741038 genotype of the *GCK* gene and dietary intake of carbohydrates. C/C carriers with high carbohydrate intake had a greater TG decrease after omega-3 supplementation compared to C/C carriers with a low carbohydrate intake, and compared to the other genotypes independent of carbohydrate intake.
Bouchard-Mercier et al., 2014	[48]	The TG response after omega-3 supplementation was dependent on interactions between dietary fat intake and SNPs within *RXRA* (rs11185660, rs10881576 and rs12339187) and *ACOX1* (rs17583163).
**Epigenetic associations with TG response to omega-3**		
Tremblay et al., 2017	[49]	The TG response after omega-3 supplementation was correlated with changes in methylation levels within *AKT3*, *ATF1*, *HDAC4*, and *IGFBP5.*
**Gene expression associations with TG respose to omega-3**		
Rundblad et al., 2019	[31]	In total 454 transcripts were differentially altered after omega-3 supplementation in TG responders to omega-3 supplementation compared to non-responders. These transcripts were related to development, immune function and lysophosphatidic acid signaling.
Rudkowska et al., 2013	[30]	TG responders to omega-3 supplementation had 252 differentially expressed transcripts after omega-3 supplementation, while non-responders had 1020 differentially expressed transcripts, and ten of these overlapped. Transcripts related to sphingolipid metabolism were altered in non-responders, and the expression of several genes related to lipid metabolism were altered in opposite directions in responders and non-responders.
**Non-coding RNA associations with TG response to omega-3**		
Ortega et al., 2015	[50]	Plasma concentration of omega-3 fatty acids increased after a diet enriched with PUFA from nuts. The change in miR-106a expression correlated with both the change in plasma EPA and TG levels.
**Gut microbiota associations with TG response to omega-3**		
Vijay et al., 2021	[51]	Supplementation with omega-3 fatty increased *Coprococcus* spp. and this change negatively correlated with the VLDL and VLDL-TG response after omega-3 supplementation.
Miao et al., 2022	[36]	The *CD36* rs1527483 variant interacted with the change in diversity and levels of *Dorea* and *Coriobacteriales Incertae Sedis* spp*,* and TG response after omega-3 supplementation.
**Habitual diet associations with TG response to omega-3**		
Rudkowska et al., 2013	[30]	DHA in red blood cells increased after omega-3 supplementation in TG responders to omega-3 supplementation, but not in non-responders.
Rundblad et al., 2019	[31]	The baseline estimated omega-3 index, reflecting habitual omega-3 intake, was lower in TG responders to omega-3 supplementation than in non-responders.

Abbreviations are explained in the list of abbreviations.

In the same study, genetic risk scores (GRS) were generated using different numbers of SNPs [42,44]. The GRS that included the most SNPs associated with the TG response to omega-3 supplementation explained 50% of the TG variability. In comparison, the same GRS only explained about 4% of the individual variation in the FINGEN study performed in the UK [44]. These differences may be due to different allelic frequencies in loci associated with the TG response between the populations in these two studies. In a double-blind, controlled, cross-over trial, the same GRS could predict the TG response to supplementation with EPA but not DHA [45].

Finally, studies have found SNPs associated with TG response to omega-3 supplementation that interact with dietary intake. Carriers of the Ala12 allele of the peroxisome proliferator activated receptor gamma (*PPARG*) gene had a more significant TG reduction after omega-3 intake than the other allele when intake of total fat and saturated fatty acids (SFA) were below 37 energy % (E%) and 10 E%, respectively [46]. Lastly, in the Canadian study, retinoid X receptor alpha (*RXRA*) and acyl-CoA oxidase 1 (*ACOX1*) SNP associations with TG response to omega-3 supplementation depended on intake of fat, and an SNP in glucokinase (*GCK*) was associated with TG response to omega-3 supplementation if the dietary intake of carbohydrates was high [47,48].

## 3 Epigenetics and TG response to omega-3 fatty acid supplementation

Epigenetics is defined as a non-heritable change in gene expression caused by, i.e., histone deacetylation by histone deacetylases (HDACs) or DNA methylation, but without changes in the DNA sequences. One study has found DNA methylation patterns associated with the TG response after intake of omega-3 fatty acids (Table 1). After a six-week omega-3 intervention, the change in DNA methylation levels in insulin like growth factor binding protein 5 (*IGFBP5*) and activating transcription factor 1 (*ATF1*) correlated with the TG response. In the same study, the methylation change in *ATF1* was also associated with the change in mRNA expression of this gene; however, the expression change did not correlate with the TG response [49].

Although few studies have investigated methylation patterns associated with the TG response to omega-3 supplementation, some studies have linked associations between omega-3 supplementation and epigenetic profiles to lipid metabolism and CVD [[52], [53], [54], [55], [56]]. For instance, a genome-wide methylation analysis revealed that omega-3 supplementation largely hypermethylated DNA, and omega-3 supplementation altered methylation of the gene encoding HDAC4, which in turn may affect histone acetylation, demonstrating the complexity of epigenetic control of gene expression [49]. Moreover, a genome-wide association study that found SNPs associated with the TG response after omega-3 supplementation also found that baseline methylation levels were associated with these SNPs and with baseline TG levels. However, no associations between methylation levels and the TG response were found [57]. Finally, omega-3 supplementation alters methylation levels in lipid metabolism-related genes in peripheral blood mononuclear cells (PBMCs). Hence, omega-3 fatty acid's effects on lipid metabolism may partly be a result of epigenetic changes [56]. Although it is not the case for all omega-3 fatty acid target genes [55], epigenetic changes after omega-3 supplementation may result in gene expression changes that may cause beneficial downstream effects.

## 4 Gene expression profiling and TG response to omega-3 fatty acid supplementation

PBMCs that include monocytes and lymphocytes, are a sound model system to understand how environmental factors such as diet can be linked to CVD progression in humans, as these cells are involved in the early development of atherosclerosis [58]. Compared to fenofibrate, which also lowers TG levels through modifying gene expression via activation of PPARs, omega-3 supplementation activates several different pathways and induces more moderate gene expression changes in humans [59]. In general, fish oil supplementation alters expression levels of genes involved in inflammatory pathways, oxidative stress response, cell cycle, cell adhesion, apoptosis, scavenger receptor activity, and DNA damage [[60], [61], [62], [63], [64]].

We have studied PBMC gene expression profiles in TG responders and non-responders to understand why some respond better to fish oil intervention than others. In this study, about 900 transcripts differed at baseline and 450 transcripts were altered differently after omega-3 supplementation between responders and non-responders. These transcripts were overrepresented in pathways involved in development, immune function and lysophosphatidic acid signaling [31] (Table 1). These findings support results from a study by Rudkowska and colleagues, who found that about 1000 and 250 transcripts were differentially expressed after omega-3 supplementation within non-responders and responders, respectively. However, only 10 of these transcripts were differentially expressed in both groups [30]. The transcriptomic analyses revealed that changes in glycerophospholipid metabolism occurred in both non-responders and responders, while gene transcripts related to sphingolipid metabolism were altered in non-responders only. These differences in transcriptomic profiles may partially account for some of the omega-3-induced TG response variation and can shed further light on the biological mechanisms of omega-3 supplementation.

## 5 Non-coding RNA and TG response to omega-3 fatty acid supplementation

Non-coding RNA includes microRNA (miRNA/miR), small interfering RNA (siRNA), Piwi-interacting RNA (piRNA) and long non-coding RNA (lncRNA) and is transcribed from DNA but not translated into proteins. Non-coding RNA regulates gene expression at the transcriptional and post-transcriptional level, such as miRNA that represses their target genes by binding to and inhibiting mRNA translation. Some miRNAs may be implicated in the TG response to omega-3 supplementation (Table 1). In an 8-week RCT where healthy women consumed 30 g/day of walnuts and almonds, plasma concentrations of both EPA and DHA increased, probably due to increased intake of alpha-linolenic acid, which can be converted to EPA and DHA. Furthermore, the concentration of 11 circulating miRNAs was altered during the intervention, including miR-106a, which correlated with the change in plasma EPA levels and TG levels [50]. miR-106a has recently been suggested to play a role in the development of atherosclerosis [65]. Moreover, miR-122 and miR-33 are important regulators of lipid metabolism, controlling the expression of genes in fatty acid and cholesterol metabolism, including fatty acid synthase (*FAS*), sterol regulatory element binding transcription factor 1 (*SREBF1*), ATP binding cassette subfamily A member 1 (*ABCA1*), ATP binding cassette subfamily G member 1 (*ABCG1*) and carnitine palmitoyltransferase 1A (*CPT1A*) [66,67]. Rats fed a cafeteria diet increased plasma TG levels and hepatic expression of miR-33a and miR-122 and consistently altered mRNA expression of these miRNAs’ target genes. Adding DHA to the diet prevented these effects; however, TG levels were not significantly reduced [66]. Interestingly, the hepatic expression change in miR-33 was reflected in PBMCs [66], suggesting that it may be possible to study lipid metabolism-related miRNAs and interactions with the TG response to omega-3 supplementation in human PBMCs.

## 6 Gut microbiota, habitual omega-3 intake, and TG response to omega-3 fatty acid supplementation

The human gut microbiota is a bacterial community of trillions of bacteria forming a complex ecosystem with the host. Omega-3 fatty acids may affect the gut microbiota by altering the composition of bacterial communities and affecting the level of inflammatory mediators and short-chain fatty acids in the gut. Conversely, the gut microbiota may affect the metabolism and absorption of omega-3 fatty acids [68]. Supplementation with omega-3 fatty acids has been shown to increase *Coprococcus* spp. and *Bacteroides* spp and decrease *Collinsella* spp*,* a genus associated with fatty liver. Moreover, *Coprococcus* spp was negatively associated with VLDL and VLDL-TG, suggesting that the gut microbiome may modulate the TG-reducing effect of omega-3 supplementation [51] (Table 1). A cohort study found a negative association between omega-3 exposure and plasma TG levels in carriers of a *CD36* genetic variant. The same SNP also interacted with associations between DHA exposure and microbial features such as higher phylogenetic diversity and higher levels of *Dorea* and *Coriobacteriales Incertae Sedis* spp [36]. Finally, omega-3 supplementation has been shown to decrease microbial diversity and increased *Bifidobacteria*. In the same study, changes in the abundance of *Atopobium* correlated with postprandial TG; however, this was not dependent on omega-3 supplementation [69].

The background diet may also affect the TG response to omega-3 supplementation [29] (Table 1). The habitual intake of omega-3 fatty acids can be estimated by the omega-3 index, which is the omega-3 content in red blood cells. A high habitual omega-3 intake may limit the additional benefit of interventions with omega-3 fatty acids. Rudkowska et al. report that the content of DHA in red blood cells increased in TG responders but not in non-responders to omega-3 supplementation [30]. In line with this, we found that TG responders to omega-3 supplementation had significantly lower baseline estimated omega-3 index than non-responders [31].

## 7 Discussion

The inter-individual TG response to omega-3 supplementation is dependent on several different factors. Genetic polymorphisms may explain much of the TG response variability [44]. SNPs may generally affect protein function if the SNP is in exons. On the other hand, if the SNP is in a regulatory sequence of a protein, it may affect the binding affinity of transcription factors. Because the activity and nuclear abundance of transcription factors such as PPARs, NFKB and SREBP are influenced by omega-3 fatty acids [23,70,71], this may be a possible mechanistic link between SNPs, omega-3 supplementation and downstream effects on TG levels. However, SNPs in lipid-metabolism-related genes associated with TG response did not seem to have a regulatory impact [[39], [40], [41],^48^]. On the other hand, a pathway analysis of genes in a GRS for the TG response to omega-3 supplementation that included 13 different loci showed that these loci were related to PPAR signaling [42]. Nonetheless, a GRS that included 31 SNPs did not include SNPs in genes that have a known role in lipid metabolism [44]. This may be explained by linkage disequilibrium between SNPs in the GRS and other causal SNPs. More knowledge about the functional effects of SNPs associated with the TG response to omega-3 supplementation is needed. This may also contribute to a better understanding of the molecular mechanisms behind the TG-reducing effect of omega-3 fatty acids.

This review shows that omega-3 fatty acids can alter methylation levels, histone modification, and miRNA and mRNA levels. Several of these changes may be related to CVD and CVD risk factors. These factors may act together with omega-3 fatty acids to fine-tune gene expression to potentially alter plasma TG levels; however, a causal link from omega-3 intake to altered epigenetic and RNA expression profiles and, further on to CVD risk factors needs to be established in future studies.

Several factors highlighted in the discussion of the contradicting results of RCTs investigating the CVD-reducing effect of omega-3 fatty acids are also relevant to the TG response [8]. Firstly, individuals with high baseline TG levels have the greatest potential for both TG reduction (Fig. 2 B) and CVD risk reduction by omega-3 fatty acids [12,72]. Secondly, the TG-reducing effect of omega-3 fatty acids is dose-dependent, and the varying omega-3 doses have been suggested as a major factor for the inconsistent results for CVD risk reduction [73,74]. Moreover, individuals with a low habitual dietary intake of omega-3 fatty acids may have a more significant TG reduction as well as CVD risk reduction following omega-3 supplementation [10,31]. This may be because of increased biologically available omega-3 fatty acids in cell membranes following supplementation. Within the cell membrane, omega-3 fatty acids may impact the formation of lipid rafts and act as substrates to produce lipid mediators [75]. Furthermore, biologically available EPA and DHA can affect the expression of genes related to lipid metabolism and inflammation and thereby reduce CVD risk [24]. Finally, when comparing results from different studies, the bioavailability and bioactivity of different chemical forms of omega-3 fatty acids are important factors to consider, as well as the common and specific effects of EPA and DHA [8,76].

## 8 Conclusion

In conclusion, the TG response after omega-3 supplementation depends on genetic variants, epigenetic and gene expression profiles, gut microbiota and the habitual intake of omega-3 fatty acids. More knowledge is needed on the mechanisms behind these interactions to understand better the current controversies of omega-3 fatty acids’ CVD risk reduction. Finally, a better understanding of these interactions may guide future personalized guidelines for omega-3 fatty acid recommendations to prevent CVD.

## Funding

This project has received funding from the European Union's Horizon 2020 research and innovation programme under grant agreement No.874739, the European Union's Horizon 2020 Research and Innovation programme under the Marie Skłodowska-Curie Actions Grant agreement No 801133, and Throne Holst Nutrition Research Foundation, Norway.

## Declaration of competing interest

The authors declare the following financial interests/personal relationships which may be considered as potential competing interests: Kirsten B Holven reports grants the three last 3 from Amgen, Sanofi, Kaneka and personal fees from Amgen, Sanofi, Pronova, outside the submitted work.

## Data Availability

No data was used for the research described in the article.

## Abbreviations

*ABCA1*: ATP binding cassette subfamily A member 1
*ABCG1*: ATP binding cassette subfamily G member 1
*ACACA*: acetyl-CoA carboxylase alpha
*ACLY*: ATP citrate lyase
*ACOX1*: acyl-CoA oxidase 1
*AGPAT4*: 1-acylglycerol-3-phosphate O-acyltransferase 4
*APOE*: apolipoprotein E
*ATF1*: activating transcription factor 1
*CD36*: CD36 molecule
CHD: coronary heart disease
*CPT1A*: carnitine palmitoyltransferase 1A
CVD: cardiovascular disease
DGAT: diacylglycerol O-acyltransferase
DHA: docosahexaenoic acid
E%: energy %
EPA: eicosapentaenoic acid
FADS: fatty acid desaturase
*FASN*: fatty acid synthase
*GCK*: glucokinase
*GPAM*: glycerol-3-phosphate acyltransferase, mitochondrial
GRS: genetic risk score
HDAC: histone deacetylase
*IGFBP5*: insulin like growth factor binding protein 5
*IQCJ-SCHIP1*: IQCJ-SCHIP1 readthrough
JADE1: jade family PHD finger 1
LC-PUFA: long chain polyunsaturated fatty acids
LDL: low-density lipoprotein
lncRNA: long non-coding RNA
*LTA*: lymphotoxin alpha
miRNA/miR: microRNA
*MYB*: MYB proto-oncogene, transcription factor
NEFA: non-esterified fatty acids
*NELL1*: neural EGFL like 1
NFE2L2: NFE2 like bZIP transcription factor 2
NFKB: nuclear factor kappa B
*NOS3*: nitric oxide synthase 3
*NXPH1*: neurexophilin 1
PBMC: peripheral blood mononuclear cell
piRNA: piwi-interacting RNA
*PLA2G4A*: phospholipase A2 group IVA
*PLA2G7*: phospholipase A2 group VII
PPAR: peroxisome proliferator-activated receptor
PPARG: peroxisome proliferator activated receptor gamma
*PUFA*: polyunsaturated fatty acids
RCT: randomized controlled trial
*RXRA*: retinoid X receptor alpha
*SCD*: stearoyl-CoA desaturase
SFA: saturated fatty acids
siRNA: small interfering RNA
*SLIT2*: slit guidance ligand 2
SNP: single-nucleotide polymorphisms
SNV: single nucleotide variants
*SREBF1*: sterol regulatory element binding transcription factor 1
SREBP: sterol regulatory binding protein
TG: triglyceride
VLDL: very low-density lipoprotein
